# Academic involution behavior, perceived academic involution climate, and English learning intention among college students: the indirect role of psychological distress

**DOI:** 10.3389/fpsyg.2026.1943950

**Published:** 2026-09-21

**Authors:** Renlong Jiang, Xinglan Zhang, Zhenzhen Li, Yandi Fu, Juan Xu, Jin Chen, Qian Ran, Xiaoxia Zhang, Tengfei Niu

**Affiliations:** 1 School of Foreign Languages, Qiannan Normal University for Nationalities, Duyun, Guizhou, China; 2 The First Clinical College, Chongqing Medical University, Chongqing, China; 3 The Second Clinical College, Chongqing Medical University, Chongqing, China; 4 School of Paediatric, Chongqing Medical University, Chongqing, China; 5 Department of Basic Courses, Chongqing Medical and Pharmaceutical College, Chongqing, China

**Keywords:** academic involution behavior, perceived academic involution climate, psychological distress, English learning intention, inconsistent mediation, college students

## Abstract

**Background:**

Academic involution may be experienced both as students' own excessive competitive behavior and as their perception that peers engage in such behavior. This study examined how academic involution behavior and perceived academic involution climate were concurrently associated with English learning intention, and whether psychological distress formed a negative statistical indirect association within the proposed model.

**Methods:**

A cross-sectional online survey was completed by 1,683 students from nine universities in Chongqing, China. Academic involution behavior, perceived academic involution climate, psychological distress, and English learning intention were assessed using four self-report instruments; the English learning intention instrument was a researcher-developed contextual brief measure. A four-factor structural equation model was estimated using diagonally weighted least squares, with age, gender, and education level included as covariates. Five direct-association hypotheses and two negative statistical indirect-association hypotheses were evaluated.

**Results:**

Academic involution behavior was positively associated with psychological distress (β = 0.207) and English learning intention (β = 0.284), and perceived academic involution climate was positively associated with psychological distress (β = 0.162) and English learning intention (β = 0.137). Psychological distress was negatively associated with English learning intention (β = −0.356; all focal paths *p* < 0.001). The standardized statistical indirect associations through psychological distress were −0.074 for academic involution behavior and −0.058 for perceived academic involution climate. The model accounted for 10.8% of the variance in psychological distress and 21.2% of the variance in English learning intention.

**Conclusion:**

The findings show contemporaneous positive direct associations alongside smaller negative statistical indirect associations, a pattern consistent with the proposed inconsistent mediation model. Because the data were cross-sectional and self-reported, the results do not establish temporal ordering, causal mediation, or intervention effects.

## 1 Introduction

English competence is important for university students‘ academic communication, postgraduate preparation, and employment, but the present outcome is not competence itself. English learning intention refers here to a student's stated readiness to undertake specified English-learning activities in the near future. It is related to, but conceptually distinct from, learning motivation, engagement, actual learning behavior, and proficiency. Evidence from Chinese university students indicates that English learning intention is associated with subsequent learning behavior, while attitudes and institutional support may condition that relationship (Yu et al., 2025). A precise focus on intention is therefore useful for examining students' readiness to initiate learning activities without assuming that intention is identical to sustained participation or achievement.

The Theory of Planned Behavior provides the conceptual basis for treating intention as a proximal antecedent of behavior (Ajzen, 1991). It also emphasizes correspondence among the action, target, context, and time specified in an intention measure (Ajzen, 2006). In this study, English learning intention is operationalized as overall readiness to study regularly, register for an English examination or competition, make a learning plan, and participate in English-practice activities. Because these items involve partly different actions and time frames, the construct is interpreted as a contextual indicator of general behavioral readiness rather than as a strict intention measure for one narrowly defined action. This boundary is important when interpreting its associations with academic competition and psychological distress.

Academic involution describes more than ordinary academic competition. It refers to an escalating pattern in which students continually increase effort to secure scarce educational advantages even as the marginal return on that effort diminishes. Contemporary scale-development research identifies excessive comparison, futile effort, hypercompetition, and related patterns as characteristic features of academic involution (Wang et al., 2024; He and Liu, 2025). Ordinary competition can occur without inefficient escalation, academic pressure refers to perceived demands rather than the behavior used to respond to them, and overwork does not necessarily arise from social comparison. Maintaining these distinctions prevents academic involution from becoming an umbrella label for every demanding educational experience.

The present study further distinguishes academic involution behavior from perceived academic involution climate. Academic involution behavior concerns a student's own excessive competitive actions, such as repetitive investment or participation undertaken to avoid falling behind. Perceived academic involution climate concerns the same student's perception that peers commonly engage in such actions and that comparison is prevalent in the learning environment (Zhou et al., 2022; Liu et al., 2024). The climate measure was not aggregated across students or modeled at the university level. It therefore represents an individual perception of the social environment, not an objective campus characteristic. Examining the two constructs together allows their associations to be compared while preserving the level at which they were measured.

A positive contemporaneous association between academic involution and English learning intention is theoretically plausible even though involution entails excessive or inefficient effort. In competitive higher-education settings, English examinations, certificates, postgraduate admission, and employment may be treated as positional resources. Students who intensify their competitive preparation, or who perceive that peers are doing so, may consequently report stronger plans to study, register for examinations, or join English-learning activities. Research on positional competition among Chinese university students describes differentiated strategies for pursuing educational advantage (Mulvey and Wright, 2022), and a study of English majors in Beijing and Macau found that involution was positively related to learning motivation while anxiety was negatively related to it (Sukjairungwattana et al., 2025). These findings support the plausibility of a positive association, but the specific roles of examination pressure, grade-point competition, postgraduate plans, employment concerns, and instrumental goals were not measured in the present study.

The same competitive escalation may also be associated with poorer psychological functioning. Individual involution behavior has been linked to anxiety, although different forms of involution do not show identical associations (Yi et al., 2022). Perceived academic involution climate has been associated with stress responses and mental exhaustion (Liu et al., 2024; Ni et al., 2024), and other cross-sectional studies have linked academic involution with mental internal friction, burnout, anxiety, and depressive symptoms (Liu et al., 2025; Li et al., 2026; Yang et al., 2026). Reverse or reciprocal patterns are also possible: anxiety and perceived stress have been associated with stronger reports of involution behavior in cross-sectional path models (Yan et al., 2022). Accordingly, the present hypotheses concern contemporaneous associations and do not assume a verified temporal sequence.

Psychological distress is relevant to English learning intention, but it must be distinguished from foreign-language emotions. The DASS-21 assesses general symptoms of depression, anxiety, and stress rather than language-specific anxiety, enjoyment, or boredom (Lovibond and Lovibond, 1995). Evidence among Chinese college students supports use of the DASS-21 as a measure of psychological distress, while retaining its depression, anxiety, and stress dimensions (Cao et al., 2023). By contrast, foreign-language emotion research examines experiences arising within language-learning contexts (Plonsky et al., 2022; Resnik et al., 2025). Both literatures are relevant, but the constructs are not interchangeable. General distress may be associated with reduced confidence, concentration, and readiness to organize learning activities; consistent with that possibility, language-learning anxiety has been linked to English-learning burnout, and broader anxiety symptoms have been associated with English academic writing through motivation and metacognitive strategies (Zheng et al., 2024; Xu et al., 2025). These processes remain possible explanations rather than variables tested in the present model.

The challenge-hindrance stressor framework provides an integrative rationale for the expected pattern. The framework originated in research distinguishing demands that may be associated with goal attainment from demands that constrain growth or achievement (Cavanaugh et al., 2000; LePine et al., 2005). Applied cautiously to academic involution, competitive demands may coincide directly with stronger behavioral preparation while also being associated with psychological strain, which in turn is associated with weaker learning intention. Contemporary refinements emphasize that challenge and hindrance appraisals should be measured rather than inferred from the stressor itself (Podsakoff et al., 2023). Because this study did not assess those appraisals, the framework organizes the hypotheses but is not treated as a mechanism demonstrated by the analysis.

Previous studies have usually addressed only part of this chain. Research on students' own involution behavior has emphasized anxiety and other mental-health outcomes (Yi et al., 2022; Liu et al., 2025), whereas research on perceived involution climate has focused on stress responses and mental exhaustion (Liu et al., 2024; Ni et al., 2024). Within English-learning contexts, prior work has examined motivation, anxiety, burnout, grit, and self-regulation more often than a clearly defined behavioral-intention construct (Wang and Zhan, 2020; Pawlak et al., 2024; Song, 2024; Sukjairungwattana et al., 2025; Zheng et al., 2024). Few studies have considered academic involution behavior and perceived academic involution climate simultaneously, separated them at the measurement level, and examined whether a positive direct association with English learning intention coexists with a negative statistical indirect association through general psychological distress.

To address this gap, we analyzed cross-sectional survey data from college students at nine universities in Chongqing, China. Structural equation modeling was used to examine the contemporaneous associations of academic involution behavior and perceived academic involution climate with psychological distress and English learning intention. We expected each involution construct to be positively associated with English learning intention and psychological distress, psychological distress to be negatively associated with English learning intention, and the two statistical indirect associations through psychological distress to be negative. Thus, the model anticipates an inconsistent mediation pattern in which positive direct associations coexist with smaller negative indirect associations. The following hypotheses are shown in Figure 1:

**Figure 1:**
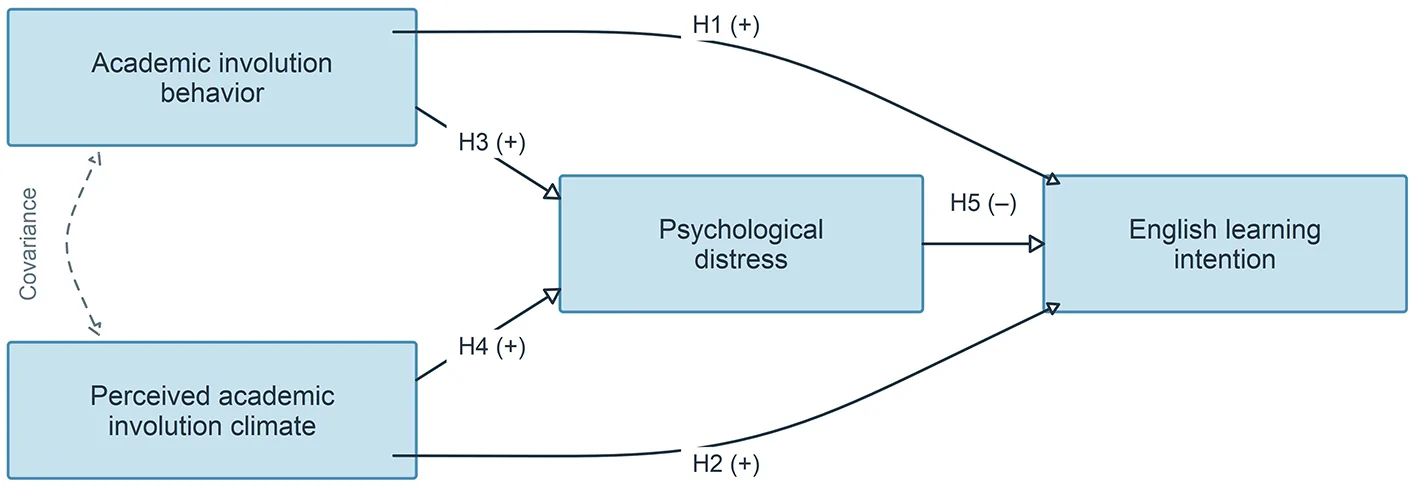
Hypothesized associational model. Solid single-headed arrows represent the five hypothesized direct associations (H1–H5), and the dashed double-headed arrow represents the covariance between academic involution behavior and perceived academic involution climate. H6 and H7 specify negative statistical indirect associations through psychological distress, represented by the corresponding two-segment solid paths. The figure is conceptual and does not imply causality.

H1. Academic involution behavior is positively associated with English learning intention.

H2. Perceived academic involution climate is positively associated with English learning intention.

H3. Academic involution behavior is positively associated with psychological distress.

H4. Perceived academic involution climate is positively associated with psychological distress.

H5. Psychological distress is negatively associated with English learning intention.

H6. Academic involution behavior has a negative statistical indirect association with English learning intention through psychological distress.

H7. Perceived academic involution climate has a negative statistical indirect association with English learning intention through psychological distress.

## 2 Materials and methods

### 2.1 Participants and procedure

This cross-sectional online survey was conducted from May 6 to 29, 2025. Students from nine universities in Chongqing were recruited by convenience sampling through Questionnaire Star. Teaching staff at participating institutions assisted only by forwarding the study information and survey link; they did not select participants, observe completion, or access individual responses. Participation was voluntary, no monetary, course-credit, or other incentives were offered, and electronic informed consent was obtained on the survey landing page. Eligibility required current enrollment at a participating institution, ability to read Chinese and complete the online questionnaire independently, and consent to participate. The survey did not include interviews and did not assess major physical illness, chronic disease, recent trauma, or severe psychiatric or cognitive impairment; these conditions were therefore not used as exclusion criteria.

The survey export contained 1,731 submissions. During data screening, 48 records were excluded, leaving 1,683 cases for analysis. The excluded records comprised questionnaires with incomplete information or missing core-scale data (*n* = 12), completion times below the prespecified minimum threshold of [(*n* = 14), clear logical inconsistencies between responses (*n* = 7), duplicate submissions (*n* = 5), straight-line responding (*n* = 4), and other clearly invalid response patterns (*n* = 6). These categories accounted for all 48 excluded records. Criteria that were not actually applied during data cleaning were not retrospectively added as exclusion criteria. University-specific recruitment counts are reported in Supplementary Table 1.

### 2.2 Measurement

#### 2.2.1 Basic demographic variables

We recorded age, gender, university, education level, current academic year, major, residence, English-learning history, academic performance, only-child status, parental education and occupation, and household income. Age, gender (0 = male, 1 = female), and education level (1 = vocational, 2 = associate, 3 = bachelor, 4 = master, 5 = doctorate) were included as covariates in the primary structural model.

#### 2.2.2 English learning intention

English learning intention was measured with a four-item researcher-developed contextual brief instrument. The items asked about studying English for at least 30 min daily during the next month, registering for an English examination or competition, developing a detailed plan within 2 weeks, and participating in an English-practice activity weekly. Responses ranged from 1 (very unlikely) to 7 (very likely); higher scores indicated stronger overall behavioral readiness. Initial items were informed by behavioral-intention and English-learning literature and adapted to the university context. Two experts with backgrounds spanning foreign-language education, educational psychology, and psychometrics qualitatively reviewed relevance, clarity, contextual suitability, and coverage, after which wording was revised. No formal content-validity index, cognitive interview, or independent pilot study was conducted. The Theory of Planned Behavior provided only the general concept of behavioral intention; the instrument does not strictly satisfy full action–target–context–time correspondence. In the standalone one-factor DWLS analysis, χ^2^(2) = 1.653, *p* = 0.438, CFI = 1.000, TLI = 1.000, RMSEA < 0.001, and SRMR = 0.026; standardized loadings ranged from 0.617 to 0.930, standardized Cronbach's α was 0.820, McDonald's ω and CR were 0.846, and AVE was 0.585. Exact items, descriptives, and standardized loadings are reported in Supplementary Table 2.

#### 2.2.3 Assessment of psychological distress

Psychological distress was assessed with the 21-item Depression Anxiety Stress Scales (DASS-21; Lovibond and Lovibond, 1995). Each subscale contains seven items scored from 0 to 3. Raw subscale totals range from 0 to 21 and were multiplied by two in this study, yielding scores from 0 to 42. The stress, depression, and anxiety subscales had Cronbach's α values of 0.922, 0.919, and 0.922, respectively. The three subscale scores were used as observed indicators of a broader latent psychological-distress factor representing their shared variance; this was not an item-level higher-order factor and not a simple composite sum. At the item level, a correlated three-factor model, χ^2^(186) = 1,628.947, *p* < 0.001, CFI = 0.996, TLI = 0.996, RMSEA = 0.068, and SRMR = 0.043, fit slightly better than a one-factor model, χ^2^(189) = 1,752.521, *p* < 0.001, CFI = 0.996, TLI = 0.996, RMSEA = 0.070, and SRMR = 0.045. Subscale-score correlations ranged from 0.873 to 0.892. Scores were analyzed continuously and were not used for diagnosis or clinical severity classification. Complete standardized item loadings for the correlated three-factor model are reported in Supplementary Table 3.

#### 2.2.4 Academic involution behavior and perceived academic involution climate

Academic involution behavior and perceived academic involution climate were measured with the two six-item instruments developed for Chinese university students by Zhou et al. (2022). Items were rated from 1 (strongly disagree) to 5 (strongly agree). Higher scores indicated more frequent self-reported academic involution behavior or a stronger individual perception that academic involution was prevalent among peers. In the present sample, Cronbach's α was 0.905 for academic involution behavior and 0.939 for perceived academic involution climate. Because the climate measure was not aggregated to the university level, it is described throughout as perceived academic involution climate.

### 2.3 Data analysis

Descriptive statistics and Spearman correlations were calculated for the scale scores. Normality was evaluated using indicator-level skewness and kurtosis and Mardia's multivariate skewness and kurtosis tests rather than relying on the Kolmogorov–Smirnov test. The 19 model indicators had skewness from −0.307 to 0.822 and kurtosis from −0.292 to 0.436; both Mardia tests were significant, indicating multivariate non-normality.

Common-method bias was addressed through procedural protections: no direct identifiers were collected, participation was voluntary, teaching staff only forwarded the link, no incentives were offered, and the instruments used different response formats. Harman's single-factor test was retained only as a limited diagnostic. Five factors had eigenvalues greater than 1, and the first factor explained 36.188% of the variance; this shows only that one factor did not dominate the item set and does not rule out common-method variance. No marker variable was available, and a latent method factor was not added because it would require untestable equality or loading assumptions. Same-source, same-time self-report bias remains a limitation.

A four-factor confirmatory measurement model was evaluated before the structural model. Standardized loadings, Cronbach's α, McDonald's ω, composite reliability, average variance extracted, latent correlations, the Fornell–Larcker criterion, and heterotrait–monotrait ratios were examined. Competing one- and two-factor models were compared for academic involution behavior and perceived academic involution climate. Complete factor loadings and reliability estimates are reported in Supplementary Tables 6, 7, pairwise discriminant-validity evidence in Supplementary Table 8, and competing measurement models in Supplementary Table 9.

Because the academic involution and English learning intention items used five- and seven-point ordered response scales and the data were multivariately non-normal, the primary measurement and structural models were estimated using diagonally weighted least squares based on polychoric and polyserial correlations. The DASS-21 subscale totals were treated as continuous indicators. Age, gender, and education level were included as covariates of psychological distress and English learning intention. Model fit was evaluated using χ^2^, *df*, *p*, RMSEA with its 90% confidence interval, CFI, TLI, and SRMR; GFI and NFI were reported as secondary indices. Statistical indirect associations and delta-method confidence intervals incorporated the covariance between constituent path estimates. Conventional maximum likelihood estimation was retained only as a sensitivity analysis. A further sensitivity model included eight university indicator variables as fixed effects; with only nine institutions, no university-level construct or multilevel inference was attempted. Analyses were conducted in R 4.6.1 and Python 3.12 using the semopy package.

## 3 Results

### 3.1 Participant characteristics

The final analytic sample comprised 1,683 students from nine universities. Participants were 17–36 years old (M = 19.39, SD = 1.68); 515 (30.6%) were male and 1,168 (69.4%) were female. English majors numbered 497 (29.5%), and 1,186 (70.5%) were enrolled in other majors. Most participants were enrolled in bachelor's programs (*n* = 1,135, 67.4%) and were in their first academic year (*n* = 1,313, 78.0%). University-specific sample sizes are reported in Supplementary Table 1; additional demographic characteristics are summarized in Table 1.

**Table 1:** Participant characteristics (*N* = 1,683).

Variable	Category	Overall
Age		19.39 ± 1.68
Age at which English learning started		9.22 ± 2.46
Gender	Male	515 (30.60%)
	Female	1,168 (69.40%)
Residence	Rural	597 (35.47%)
	Urban	1,086 (64.53%)
English major status	No	1,186 (70.47%)
	Yes	497 (29.53%)
Academic performance category	Top 10%	207 (12.30%)
	10%−30%	495 (29.41%)
	30%−50%	513 (30.48%)
	50%−70%	368 (21.87%)
	below top 70%	100 (5.94%)
Only child status	No	1,279 (76.00%)
	Yes	404 (24.00%)
Father's educational level	Primary school	290 (17.23%)
	junior high school	679 (40.34%)
	senior high school	421 (25.01%)
	college or above	293 (17.41%)
Father's occupation	Non-manual/white-collar occupation	378 (22.46%)
	Manual/blue-collar or unstable occupation	1,305 (77.54%)
Mother's educational level	Primary school	484 (28.76%)
	Junior high school	621 (36.90%)
	Senior high school	358 (21.27%)
	College or above	220 (13.07%)
Mother's occupation	Non-manual/white-collar occupation	388 (23.05%)
	Manual/blue-collar or unstable occupation	1295 (76.95%)
Annual household income	Low (0–50,000)	808 (48.01%)
	Middle (50,000–200,000)	715 (42.48%)
	High (>200,000)	160 (9.51%)

### 3.2 Preliminary analyses

The 19 SEM indicators showed modest univariate departures from normality, but Mardia's multivariate skewness test, $\chi_{\left(1,330\right)}^{2}$ = 12,282.849, *p* < 0.001, and multivariate kurtosis test, z = 186.274, *p* < 0.001, indicated multivariate non-normality. Harman's diagnostic extracted five factors with eigenvalues greater than 1; the first explained 36.188% of the variance. This result does not establish the absence of common-method bias. Indicator-level distributional statistics are reported in Supplementary Table 4, and estimator-fit and focal-path sensitivity results are reported in Supplementary Table 5.

### 3.3 Correlations among study variables

As shown in Table 2, academic involution behavior and perceived academic involution climate were positively correlated with English learning intention and psychological distress, whereas psychological distress was negatively correlated with English learning intention. The bivariate correlations involving psychological distress and English learning intention were small (|*r*| = 0.081–0.188), while academic involution behavior and perceived academic involution climate were moderately correlated (*r* = 0.409).

**Table 2:** Descriptive statistics and Spearman correlations among the focal variables.

Variable	English learning intention	Academic involution behavior	Perceived academic involution climate	Psychological distress
English learning intention	1.000			
Academic involution behavior	0.188^***^	1.000		
Perceived academic involution climate	0.178^***^	0.409^***^	1.000	
Psychological distress	−0.110^***^	0.083^***^	0.081^***^	1.000

*N* = 1,683. Asterisks indicate two-tailed significance: ^***^p < 0.001.

### 3.4 Measurement model evaluation

The four-factor measurement model fit the data well, χ^2^(146) = 325.162, *p* < 0.001, χ^2^/*df* = 2.227, CFI = 0.995, TLI = 0.994, RMSEA = 0.027, and SRMR = 0.072. The standardized four factor measurement model is shown in Figure 2. Standardized loadings ranged from 0.513 to 0.916; all freely estimated loadings were significant at *p* < 0.001. Across constructs, Cronbach's α ranged from 0.815 to 0.957, McDonald's ω from 0.777 to 0.953, composite reliability from 0.773 to 0.953, and average variance extracted from 0.533 to 0.773. All square roots of average variance extracted exceeded the corresponding latent correlations, and HTMT values ranged from 0.096 to 0.481. The HTMT value was 0.481 (bootstrap 95% CI [0.421, 0.534]) for academic involution behavior vs. perceived academic involution climate and 0.237 (95% CI [0.188, 0.296]) for academic involution behavior vs. English learning intention. A correlated two-factor model of the two involution constructs, χ^2^(53) = 172.340, CFI = 0.997, TLI = 0.996, RMSEA = 0.037, and SRMR = 0.035, fit markedly better than the corresponding single-factor model, χ^2^(54) = 3,738.005, CFI = 0.899, TLI = 0.877, RMSEA = 0.201, and SRMR = 0.167. These results support treating the four constructs as related but distinguishable. Complete evidence is reported in Supplementary Tables 6–9.

**Figure 2:**
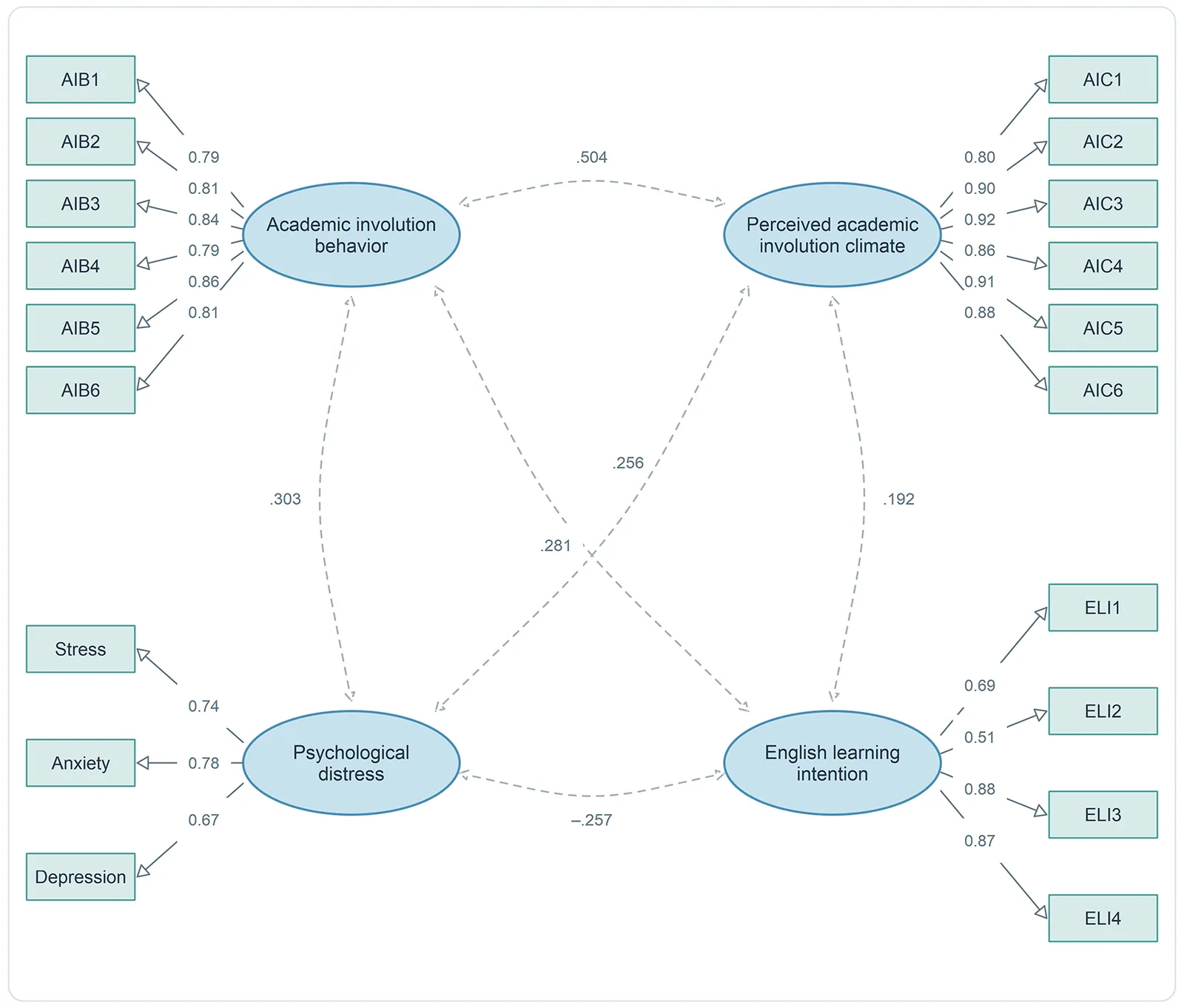
Four-factor measurement model estimated using DWLS. Values are standardized factor loadings and latent correlations. Residual variances were estimated but are omitted for readability. No structural regression paths or correlated residuals are shown.

### 3.5 Structural model fit and path estimates

The final DWLS structural model, which contained no correlated residuals, fit the data well: $\chi_{\left(194\right)}^{2}$ = 438.512, *p* < 0.001, χ^2^/*df* = 2.260, CFI = 0.994, TLI = 0.992, RMSEA = 0.027, 90% CI [0.024, 0.031], SRMR = 0.063, GFI = 0.989, and NFI = 0.989 (Table 3). The final structural model with standardized path coefficients is shown in Figure 3. These indices met the stated fit criteria. The focal paths had the same directions and significance conclusions under conventional maximum likelihood estimation (Supplementary Table 5). The nine-university fixed-effects sensitivity model also retained the directions and significance conclusions of all five focal paths and showed χ^2^(322) = 1,922.621, *p* < 0.001, χ^2^/*df* = 5.971, CFI = 0.945, TLI = 0.923, RMSEA = 0.054, and SRMR = 0.034. Its focal paths are reported in Supplementary Table 10.

**Table 3:** Fit indices for the final DWLS structural model.

Fit index	Value
χ^2^	438.512
*df*	194
*p*	< 0.001
χ^2^/*df*	2.260
CFI	0.994
TLI	0.992
RMSEA (90% CI)	0.027 [0.024, 0.031]
SRMR	0.063
GFI	0.989
NFI	0.989

The model included age, gender, and education level as covariates and contained no correlated residuals.

**Figure 3:**
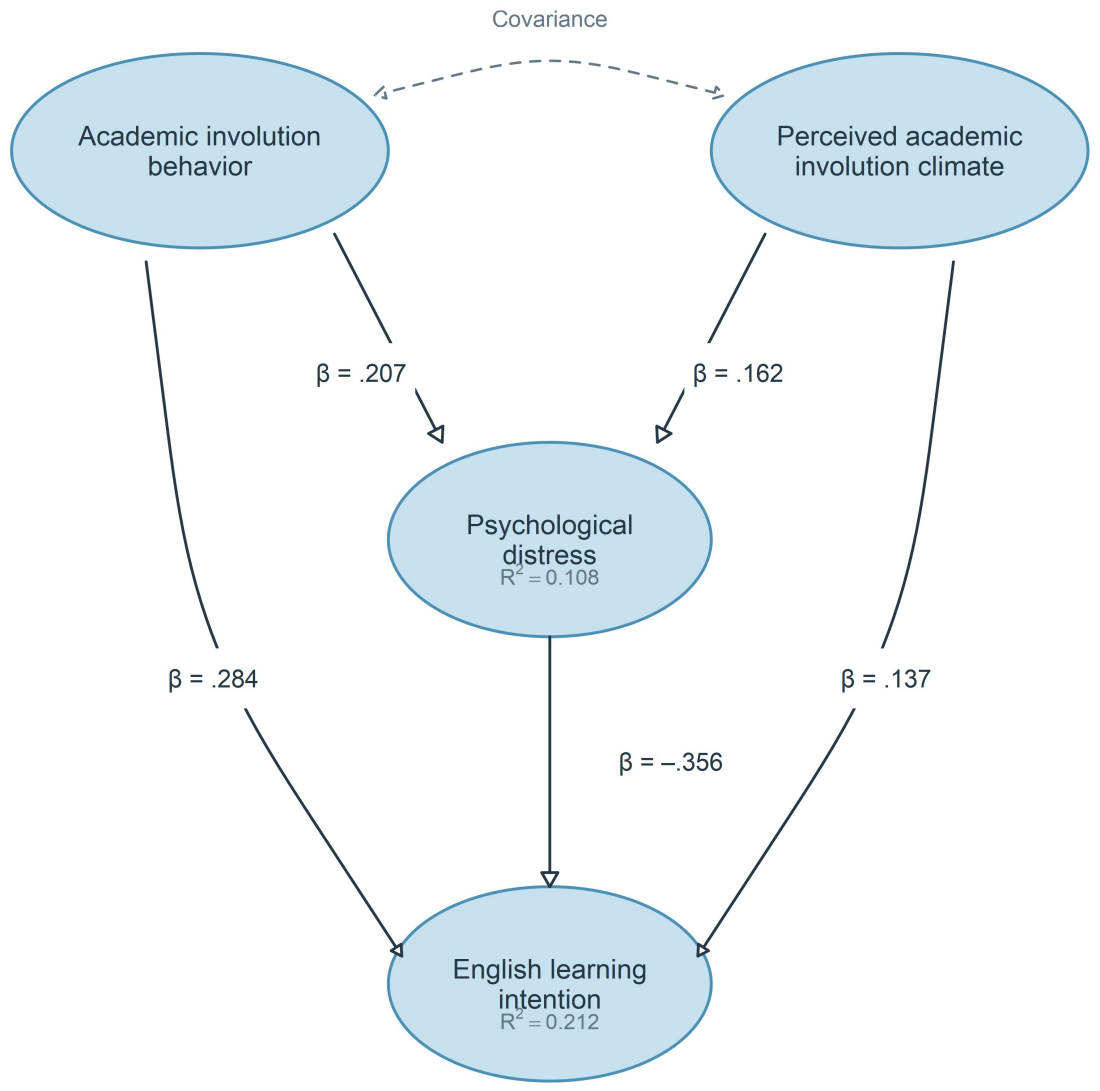
Final DWLS structural model with standardized path coefficients. The dashed double-headed arrow represents the covariance between the two exogenous latent variables. Age, gender, and education-level covariate paths were included in estimation but omitted for readability; all are reported in Table 4. The final model included no correlated residuals. *R*^2^ values are shown for endogenous latent variables.

Academic involution behavior was positively associated with psychological distress (β = 0.207, *p* < 0.001) and English learning intention (β = 0.284, *p* < 0.001). Perceived academic involution climate was also positively associated with psychological distress (β = 0.162, *p* < 0.001) and English learning intention (β = 0.137, *p* < 0.001). Psychological distress was negatively associated with English learning intention (β = −0.356, *p* < 0.001). These results support H1–H5 as contemporaneous associations. The model explained 10.8% of the variance in psychological distress and 21.2% of the variance in English learning intention. Complete unstandardized and standardized estimates, covariate paths, *p* values, and confidence intervals are reported in Table 4.

**Table 4:** Complete path estimates for the final DWLS structural model.

Predictor	Outcome	B	SE	β	z	*p*	95% CI for B
Academic involution behavior	Psychological distress	0.226	0.035	0.207	6.383	< 0.001	[0.156, 0.295]
Perceived academic involution climate	Psychological distress	0.172	0.034	0.162	5.118	< 0.001	[0.106, 0.237]
Academic involution behavior	English learning intention	0.256	0.028	0.284	9.031	< 0.001	[0.201, 0.312]
Perceived academic involution climate	English learning intention	0.121	0.026	0.137	4.658	< 0.001	[0.070, 0.172]
Psychological distress	English learning intention	−0.296	0.026	−0.356	−11.580	< 0.001	[−0.346, −0.246]
Age	Psychological distress	0.060	0.025	0.070	2.432	0.015	[0.012, 0.109]
Gender	Psychological distress	−0.047	0.022	−0.055	−2.126	0.034	[−0.091, −0.004]
Education level	Psychological distress	−0.004	0.025	−0.005	−0.157	0.876	[−0.052, 0.045]
Age	English learning intention	−0.079	0.019	−0.112	−4.168	< 0.001	[−0.117, −0.042]
Gender	English learning intention	0.040	0.017	0.057	2.359	0.018	[0.007, 0.074]
Education level	English learning intention	0.115	0.019	0.162	5.998	< 0.001	[0.077, 0.153]

Gender was coded 0 = male and 1 = female. Education level was coded from 1 = vocational to 5 = doctorate. B = unstandardized estimate; β = standardized estimate. Two-tailed *p* values are shown.

### 3.6 Statistical indirect associations

Academic involution behavior had a negative statistical indirect association with English learning intention through psychological distress (B = −0.067, SE = 0.012, β = −0.074, 95% CI [−0.090, −0.043], *p* < 0.001). The corresponding indirect association for perceived academic involution climate was also negative (B = −0.051, SE = 0.011, β = −0.058, 95% CI [−0.072, −0.030], *p* < 0.001) (Table 5). These small negative indirect associations coexisted with positive direct associations, producing an inconsistent mediation pattern and supporting H6–H7 at the level of statistical association. They do not establish temporal or causal mediation.

**Table 5:** Statistical indirect associations through psychological distress.

Exposure	Direct β	Indirect B	SE	Indirect β	z	*p*	95% CI for B	Total β
Academic involution behavior	0.284	−0.067	0.012	−0.074	−5.616	< 0.001	[−0.090, −0.043]	0.210
Perceived academic involution climate	0.137	−0.051	0.011	−0.058	−4.737	< 0.001	[−0.072, −0.030]	0.080

Delta-method standard errors and confidence intervals incorporate the covariance between constituent path estimates. Total β is the sum of the standardized direct and indirect associations. These cross-sectional indirect associations do not establish causal mediation.

## 4 Discussion

This study examined the contemporaneous relations of academic involution behavior and perceived academic involution climate with psychological distress and English learning intention. Both involution constructs had positive direct associations with English learning intention and psychological distress, whereas psychological distress was negatively associated with English learning intention. Positive direct associations therefore coexisted with smaller negative statistical indirect associations through psychological distress. The model explained 10.8% of the variance in psychological distress and 21.2% of the variance in English learning intention. The findings are internally consistent with the proposed model, but their explanatory and practical magnitude is modest.

The positive direct associations with English learning intention are compatible with an instrumental response to competitive educational conditions. Students who invest more heavily in positional competition, or who perceive such investment among peers, may also report stronger readiness to study English, make plans, register for examinations, and participate in practice activities. This interpretation is consistent with research describing credential-oriented strategies among Chinese university students (Mulvey and Wright, 2022) and with evidence that involution was positively related to motivation among English majors in Beijing and Macau (Sukjairungwattana et al., 2025). However, motivation and intention are not equivalent, and the present study did not measure examination pressure, grade-point competition, postgraduate plans, employment concerns, credential goals, or students' challenge appraisals. These factors should therefore be treated as plausible contextual explanations rather than conclusions directly supported by the data. The single measurement occasion also provides no basis for describing the association as short-term or long-term.

The two involution constructs contributed related but non-identical information. Academic involution behavior represented respondents' own competitive escalation, whereas perceived academic involution climate represented their view of what peers commonly did. Their moderate correlation and the measurement-model comparisons supported treating them as distinguishable latent constructs, including clear discriminant validity between academic involution behavior and English learning intention. At the same time, both involution constructs were reported by the same students at the same time. The observed distinctions therefore do not transform perceived climate into an objective university-level exposure, and common-method variance may have contributed to their associations. The nine-university fixed-effects sensitivity analysis controlled mean differences among the sampled institutions but did not provide a multilevel test of campus climate.

The positive associations with psychological distress accord with a broader literature on the psychological costs of academic involution. Individual involution behavior has shown heterogeneous associations with anxiety depending on whether the behavior is passive, reward-oriented, or achievement-motivated (Yi et al., 2022). Perceived involution climate has been associated with stress responses and mental exhaustion (Liu et al., 2024; Ni et al., 2024), while academic involution has also been linked to academic stress and mental internal friction, burnout, anxiety, and depressive symptoms (Liu et al., 2025; Li et al., 2026; Yang et al., 2026). These converging findings make a positive contemporaneous association plausible. Nevertheless, they do not establish that involution preceded distress. Distressed students may perceive competition more negatively or engage in more involution behavior, and other conditions may influence both constructs.

Psychological distress was negatively associated with English learning intention. General symptoms of depression, anxiety, and stress may accompany diminished concentration, confidence, and readiness to organize effort, but these specific processes were not measured. The result should also not be equated with evidence on foreign-language anxiety or enjoyment, which are domain-specific experiences (Plonsky et al., 2022; Resnik et al., 2025). Studies of English learning nevertheless provide relevant contextual comparisons: language-learning anxiety has been associated with burnout (Zheng et al., 2024), and broader anxiety symptoms have been associated with academic writing performance through motivation and metacognitive strategies (Xu et al., 2025). Positive and negative emotions, perceived competence, grit, and self-regulation are also related to motivated language-learning behavior (Wang and Zhan, 2020; Pawlak et al., 2024; Song, 2024; Zeidner and Stoeger, 2019). In the present study, however, self-regulated learning and these domain-specific emotions were not assessed and cannot be presented as tested mechanisms.

The opposite signs of the direct and indirect associations form an inconsistent mediation pattern. The challenge-hindrance stressor framework helps make this pattern conceptually coherent: a competitive demand may be associated with greater goal-directed preparation while simultaneously being associated with strain (Cavanaugh et al., 2000; LePine et al., 2005). Yet the framework does not allow a researcher to label a demand as a challenge or hindrance without measuring how participants appraise it (Podsakoff et al., 2023). The present results are therefore consistent with, but do not verify, an appraisal-based explanation. Similarly, person-environment fit, self-regulated learning, and control-value processes were not directly measured. They may guide future tests, but they should not be treated as causal processes established by the structural model.

The statistical significance of the model should be interpreted alongside effect magnitude. Several bivariate correlations involving psychological distress and English learning intention were weak, and the two standardized indirect associations were small (β = –0.074 and −0.058). The large sample increased the precision with which these associations were estimated. Although the model accounted for 21.2% of English learning intention, most variance remained unexplained, and only 10.8% of psychological distress was explained. These results do not support broad claims that academic involution has a powerful dual effect or that reducing psychological distress alone would substantially change English learning intention. They instead identify a modest coexistence of positive direct and negative indirect statistical associations that warrants replication.

The study's principal contribution is to connect previously separated lines of research without collapsing their constructs. It distinguishes students' own involution behavior from their individually perceived involution climate, defines English learning intention separately from motivation and engagement, and treats DASS-21 scores as general psychological-distress symptoms rather than foreign-language emotions or clinical diagnoses. This structure extends prior work on involution and mental health (Liu et al., 2024; Ni et al., 2024; Yi et al., 2022) and on anxiety, motivation, and burnout in English learning (Pawlak et al., 2024; Sukjairungwattana et al., 2025; Zheng et al., 2024). Its contribution lies in the combined associational pattern, not in evidence for temporal mediation or a universal motivational mechanism.

The findings support restrained practical considerations. University English teaching need not rely on rankings, repeated examinations, or peer comparison as the principal basis for engagement. Clear learning objectives, formative feedback, achievable practice opportunities, and links between English learning, authentic communication, and professional development may help students pursue purposeful learning without intensifying competitive pressure. Universities may also make psychological support accessible to students who report persistent distress. These proposals are consistent with the observed associations, but they are not intervention effects demonstrated by the present study. Their effectiveness should be evaluated directly before they are presented as evidence-based solutions.

Future research can test the interpretations that the present design leaves unresolved. Multi-wave studies could examine whether involution behavior and perceived climate precede changes in distress and intention, whether the reverse sequence is also present, and whether the positive and negative paths operate over different time scales. Studies should directly measure challenge and hindrance appraisals, instrumental goals, examination and employment concerns, foreign-language emotions, self-regulated learning, teacher and peer support, and actual learning behavior. Multi-source data, such as learning-platform records, classroom participation, examination performance, and teacher reports, would reduce same-source dependence. A larger number of institutions would also permit aggregation statistics and multilevel analysis of university climate rather than relying on individual perceptions and fixed-effects sensitivity checks.

## 5 Limitations and future directions

Several limitations concern temporal and causal interpretation. The single-wave cross-sectional design cannot establish whether academic involution preceded psychological distress or whether distress preceded changes in perceived competition and learning intention. Statistical indirect associations do not demonstrate causal mediation, and unmeasured confounding remains possible. Longitudinal, cross-lagged, and intervention designs are needed before temporal or causal claims can be made.

Measurement limitations also require caution. All focal variables were reported by the same students at the same time, and the limited Harman diagnostic cannot rule out common-method variance; no marker variable or multi-source measurement was available. The English learning intention instrument was a researcher-developed contextual brief measure. Although its one-factor model and reliability were acceptable, the four items refer to partly different actions and time frames, and no formal content-validity index, cognitive interview, or independent pilot study was conducted. DASS-21 scores represent self-reported symptoms rather than diagnoses, and English learning intention cannot be equated with actual learning behavior or proficiency.

The sampling and analytic scope further limit generalizability. Convenience sampling from nine universities in one municipality may not represent students in other regions, institution types, or disciplines. Nine institutions are insufficient for robust university-level inference; the fixed-effects analysis only controlled mean differences among the sampled universities. In addition, mutually exclusive exclusion-reason codes were not retained in the analytic file, so category-specific cleaning counts cannot be reconstructed. Future studies should use more diverse probability or stratified samples, preregistered cleaning rules, retained screening logs, and sufficiently many institutions for multilevel analysis.

## 6 Conclusion

Academic involution behavior and perceived academic involution climate were concurrently associated with stronger English learning intention and greater psychological distress, while psychological distress was associated with weaker English learning intention. The resulting small negative statistical indirect associations coexisted with positive direct associations. This inconsistent pattern is descriptive of the present cross-sectional model and should not be interpreted as evidence that involution causes distress or that distress causally reduces learning intention.

## Data Availability

The original contributions presented in the study are included in the article/Supplementary material, further inquiries can be directed to the corresponding author.
