# Supplementary material for: Academic involution behavior, perceived academic involution climate, and English learning intention among college students: the indirect role of psychological distress
Source: Front Psychol. 2026 Sep 21;17:1943950. doi: 10.3389/fpsyg.2026.1943950 (PMC13636433; doi:10.3389/fpsyg.2026.1943950)
Supplement: Supplementary file 1 [file Supplementary_file_1.DOCX]

**Supplementary Materials**

**Table S1. Participant distribution across the nine universities**

| **University** | **Original Chinese label** | **n** | **%** |
| --- | --- | --- | --- |
| Chongqing Medical and Pharmaceutical College | 重庆医药高等专科学校 | 492 | 29.2 |
| Chongqing University of Posts and Telecommunications | 重庆邮电大学 | 341 | 20.3 |
| Chongqing Medical University | 重庆医科大学 | 260 | 15.4 |
| Chongqing College of Traditional Chinese Medicine | 重庆中医药学院 | 162 | 9.6 |
| Sichuan International Studies University | 四川外国语大学 | 144 | 8.6 |
| Chongqing University of Arts and Sciences | 重庆文理学院 | 83 | 4.9 |
| Chongqing Institute of Foreign Studies | 重庆外语外事学院 | 78 | 4.6 |
| Chongqing Three Gorges University | 重庆三峡学院 | 73 | 4.3 |
| Chongqing University of Science and Technology | 重庆科技大学 | 50 | 3.0 |
| Total | — | 1,683 | 100.0 |

*Note. University is reported as an individual participant characteristic. No aggregation, rwg, ICC(1), ICC(2), or university-level climate inference was performed.*

**Table S2. English learning intention items, descriptives, and standardized loadings**

| **Item** | **Original Chinese wording** | **English translation** | **Time frame** | **M** | **SD** | **Std. loading** |
| --- | --- | --- | --- | --- | --- | --- |
| ELI1 | 我计划在未来一个月内每天至少学习30分钟英语 | I plan to study English for at least 30 minutes every day during the next month. | Next month / daily | 4.784 | 1.340 | 0.800 |
| ELI2 | 我打算报名参加下一次雅思/托福/四六级/其他英语类比赛考试以检验学习成果 | I intend to register for the next IELTS, TOEFL, CET, or another English-language competition or examination to assess my learning outcomes. | Next available event | 4.887 | 1.526 | 0.673 |
| ELI3 | 我会在未来两周内制定详细的英语学习计划 | I will develop a detailed English-learning plan within the next two weeks. | Next two weeks | 4.485 | 1.300 | 0.930 |
| ELI4 | 我计划每周参与一次英语实践活动（如英语角、演讲比赛、英语小组讨论等） | I plan to participate in an English-practice activity once a week, such as an English corner, speech contest, or English group discussion. | Weekly | 3.971 | 1.393 | 0.617 |

*Note. All items used a 1 (very unlikely) to 7 (very likely) response scale. The instrument is a researcher-developed contextual brief measure. Two experts qualitatively reviewed relevance, clarity, contextual suitability, and coverage. No formal content-validity index, cognitive interview, or independent pilot study was conducted. Loadings are from the standalone one-factor DWLS model.*

**Table S3. Standardized loadings for the correlated three-factor DASS-21 model**

| **Factor** | **DASS-21 item** | **Standardized loading** |
| --- | --- | --- |
| Stress | DASS-1 | 0.730 |
| Stress | DASS-6 | 0.808 |
| Stress | DASS-8 | 0.893 |
| Stress | DASS-11 | 0.916 |
| Stress | DASS-12 | 0.913 |
| Stress | DASS-14 | 0.864 |
| Stress | DASS-18 | 0.853 |
| Depression | DASS-3 | 0.818 |
| Depression | DASS-5 | 0.736 |
| Depression | DASS-10 | 0.877 |
| Depression | DASS-13 | 0.936 |
| Depression | DASS-16 | 0.887 |
| Depression | DASS-17 | 0.890 |
| Depression | DASS-21 | 0.855 |
| Anxiety | DASS-2 | 0.768 |
| Anxiety | DASS-4 | 0.865 |
| Anxiety | DASS-7 | 0.859 |
| Anxiety | DASS-9 | 0.851 |
| Anxiety | DASS-15 | 0.902 |
| Anxiety | DASS-19 | 0.886 |
| Anxiety | DASS-20 | 0.897 |

*Note. Stress, anxiety, and depression remained distinguishable item-level dimensions. DASS-21 subscale scores were analyzed continuously and were not used for diagnosis or clinical severity classification.*

**Table S4. Distributional characteristics of the 19 observed SEM indicators**

| **Indicator** | **Response range** | **M** | **SD** | **Skewness** | **Excess kurtosis** |
| --- | --- | --- | --- | --- | --- |
| a1 | 1-5 | 3.100 | 0.898 | -0.187 | -0.044 |
| a2 | 1-5 | 3.015 | 0.853 | -0.086 | 0.106 |
| a3 | 1-5 | 3.077 | 0.877 | -0.213 | 0.027 |
| a4 | 1-5 | 2.904 | 0.897 | -0.059 | -0.091 |
| a5 | 1-5 | 2.907 | 0.870 | -0.058 | 0.040 |
| a6 | 1-5 | 2.749 | 0.885 | -0.043 | -0.066 |
| b1 | 1-5 | 3.477 | 0.809 | -0.149 | -0.006 |
| b2 | 1-5 | 3.291 | 0.805 | 0.008 | 0.303 |
| b3 | 1-5 | 3.310 | 0.802 | -0.041 | 0.303 |
| b4 | 1-5 | 3.314 | 0.795 | -0.054 | 0.277 |
| b5 | 1-5 | 3.263 | 0.806 | -0.019 | 0.219 |
| b6 | 1-5 | 3.195 | 0.798 | 0.015 | 0.436 |
| stress_total | 0-42 | 11.105 | 8.785 | 0.561 | -0.292 |
| anxiety_total | 0-42 | 9.009 | 8.445 | 0.822 | 0.013 |
| depression_total | 0-42 | 9.465 | 8.487 | 0.787 | 0.002 |
| eli1 | 1-7 | 4.784 | 1.340 | -0.145 | -0.027 |
| eli2 | 1-7 | 4.887 | 1.526 | -0.307 | -0.276 |
| eli3 | 1-7 | 4.485 | 1.300 | -0.184 | 0.420 |
| eli4 | 1-7 | 3.971 | 1.393 | -0.130 | 0.081 |

*Note. Skewness ranged from −.307 to .822 and excess kurtosis from −.292 to .436. Multivariate normality results are reported in the manuscript text.*

**Table S5. Model fit and focal-path sensitivity under alternative estimators**

| **Analysis** | **Metric or path** | **Conventional ML** | **DWLS** | **Interpretation** |
| --- | --- | --- | --- | --- |
| Model fit | χ² | 1632.666 | 438.512 | DWLS retained as primary |
| Model fit | df | 194 | 194 | DWLS retained as primary |
| Model fit | χ²/df | 8.416 | 2.260 | DWLS retained as primary |
| Model fit | CFI | 0.942 | 0.994 | DWLS retained as primary |
| Model fit | TLI | 0.930 | 0.992 | DWLS retained as primary |
| Model fit | RMSEA | 0.066 | 0.027 | DWLS retained as primary |
| Model fit | SRMR | 0.042 | 0.063 | DWLS retained as primary |
| Focal path | AIB → psychological distress | β = 0.071, p =0 .019 | β = 0.207, p = < 0.001 | Same direction; significant |
| Focal path | Perceived AIC → psychological distress | β = 0.087, p = 0.003 | β = 0.162, p = < 0.001 | Same direction; significant |
| Focal path | AIB → English learning intention | β = 0.202, p = < 0.001 | β = 0.284, p = < 0.001 | Same direction; significant |
| Focal path | Perceived AIC → English learning intention | β = 0.082, p = 0.005 | β = 0.137, p = < 0.001 | Same direction; significant |
| Focal path | Psychological distress → English learning intention | β = -0.115, p = < 0.001 | β = -0.356, p = < 0.001 | Same direction; significant |

*Note. DWLS was the primary estimator. Conventional ML was retained as a sensitivity analysis. The directions and significance conclusions of all focal paths were consistent; coefficient magnitudes are not directly interchangeable across estimators.*

**Table S6. Complete factor loadings for the four-construct measurement model**

| **Construct** | **Indicator** | **B** | **SE** | **z** | **p** | **Std. loading** |
| --- | --- | --- | --- | --- | --- | --- |
| AIB | a1 | 1.000 | — | — | — | 0.786 |
| AIB | a2 | 1.025 | 0.028 | 36.194 | <0 .001 | 0.806 |
| AIB | a3 | 1.074 | 0.028 | 38.456 | <0 .001 | 0.844 |
| AIB | a4 | 1.002 | 0.028 | 35.162 | <0 .001 | 0.787 |
| AIB | a5 | 1.091 | 0.028 | 39.224 | <0 .001 | 0.857 |
| AIB | a6 | 1.028 | 0.028 | 36.364 | <0 .001 | 0.809 |
| Perceived AIC | b1 | 1.000 | — | — | — | 0.805 |
| Perceived AIC | b2 | 1.121 | 0.025 | 45.539 | <0 .001 | 0.903 |
| Perceived AIC | b3 | 1.137 | 0.024 | 46.524 | <0 .001 | 0.916 |
| Perceived AIC | b4 | 1.063 | 0.025 | 41.971 | <0 .001 | 0.856 |
| Perceived AIC | b5 | 1.129 | 0.025 | 46.005 | <0 .001 | 0.909 |
| Perceived AIC | b6 | 1.094 | 0.025 | 43.841 | <0 .001 | 0.881 |
| Psychological distress | stress_total | 1.000 | — | — | — | 0.739 |
| Psychological distress | anxiety_total | 1.106 | 0.045 | 24.353 | <0 .001 | 0.777 |
| Psychological distress | depression_total | 0.833 | 0.036 | 22.992 | <0 .001 | 0.671 |
| ELI | eli1 | 1.000 | — | — | — | 0.688 |
| ELI | eli2 | 0.745 | 0.038 | 19.409 | <0 .001 | 0.513 |
| ELI | eli3 | 1.282 | 0.041 | 31.096 | <0 .001 | 0.882 |
| ELI | eli4 | 1.266 | 0.041 | 30.964 | <0 .001 | 0.871 |

*Note. The joint four-factor model was estimated by DWLS. The first loading for each construct was fixed to 1.000 for identification; all freely estimated loadings were significant at p < .001.*

**Table S7. Reliability and convergent validity**

| **Construct** | **Cronbach's α** | **McDonald's ω** | **CR** | **AVE** |
| --- | --- | --- | --- | --- |
| Academic involution behavior | 0.905 | 0.922 | 0.922 | 0.665 |
| Perceived academic involution climate | 0.939 | 0.953 | 0.953 | 0.773 |
| Psychological distress | 0.957 | 0.777 | 0.773 | 0.533 |
| English learning intention | 0.815 | 0.835 | 0.835 | 0.569 |

*Note. Cronbach's α and McDonald's ω assess internal consistency; CR denotes composite reliability and AVE denotes average variance extracted. The standalone English learning intention analysis reports standardized α = .820, whereas this table reports the scale-level estimate used in the complete measurement assessment.*

**Table S8. Pairwise discriminant-validity evidence**

| **Construct 1** | **Construct 2** | **√AVE 1** | **√AVE 2** | **Latent r** | **HTMT** | **Bootstrap 95% CI** |
| --- | --- | --- | --- | --- | --- | --- |
| Academic involution behavior | Perceived academic involution climate | 0.815 | 0.879 | 0.504 | 0.481 | [0.421, 0.534] |
| Academic involution behavior | Psychological distress | 0.815 | 0.730 | 0.303 | 0.110 | — |
| Academic involution behavior | English learning intention | 0.815 | 0.754 | 0.256 | 0.237 | [0.188, 0.296] |
| Perceived academic involution climate | Psychological distress | 0.879 | 0.730 | 0.281 | 0.111 | — |
| Perceived academic involution climate | English learning intention | 0.879 | 0.754 | 0.192 | 0.178 | — |
| Psychological distress | English learning intention | 0.730 | 0.754 | -0.257 | 0.096 | — |

*Note. For every construct pair, each square root of AVE exceeded the absolute latent correlation. All HTMT values were below conventional cutoffs. Bootstrap confidence intervals were emphasized for the two pairs of greatest conceptual concern; neither interval included 1.*

**Table S9. Competing measurement models for academic involution behavior and perceived academic involution climate**

| **Model** | **χ² (df)** | **χ²/df** | **CFI** | **TLI** | **RMSEA** | **SRMR** |
| --- | --- | --- | --- | --- | --- | --- |
| Correlated two-factor model | 172.340 (53) | 3.252 | 0.997 | 0.996 | 0.037 | 0.035 |
| Single-factor model | 3738.005 (54) | 69.222 | 0.899 | 0.877 | 0.201 | 0.167 |

*Note. The correlated two-factor model fit markedly better than the single-factor model, supporting treatment of academic involution behavior and perceived academic involution climate as related but distinguishable constructs.*

**Table S10. Focal paths in the primary model and university fixed-effects sensitivity model**

| **Path** | **Primary DWLS β** | **p** | **ML fixed-effects β** | **p** | **Conclusion** |
| --- | --- | --- | --- | --- | --- |
| AIB → psychological distress | 0.207 | < 0.001 | 0.077 | 0.011 | Direction and significance retained |
| Perceived AIC → psychological distress | 0.162 | < 0.001 | 0.083 | 0.005 | Direction and significance retained |
| AIB → English learning intention | 0.284 | < 0.001 | 0.211 | < 0.001 | Direction and significance retained |
| Perceived AIC → English learning intention | 0.137 | < 0.001 | 0.074 | 0.013 | Direction and significance retained |
| Psychological distress → English learning intention | −0.356 | < 0.001 | −0.120 | < 0.001 | Direction and significance retained |

*Note. Eight university indicator variables were entered as fixed effects. This analysis controls mean differences among the sampled institutions but is not a multilevel analysis and does not estimate a university-level climate. The primary and sensitivity columns use different estimators and specifications, so coefficient magnitudes should not be treated as directly comparable.*
